# Integrating anatomy learning: A comparative study of anatomical digital resource and flipped model with sectra

**DOI:** 10.1371/journal.pone.0356693

**Published:** 2026-08-31

**Authors:** Aisha Abdul Haq, Sonia Ijaz Haider, Shumaila Rafi, Naheed Khan, Fareeha Butt, Dur-e-shewar Rehman

**Affiliations:** 1 Department of Anatomy, Dow Medical College, Dow University of Health Sciences, Karachi, Sind, Pakistan; 2 Dow Institute of Health Professions Education, Dow University of Health Sciences, Karachi, Sind, Pakistan; 3 Department of Physiology, Dow Medical College, Dow university of Health Sciences, Karachi, Sind, Pakistan; 4 Basic Sciences Department, College of Science and Health Professions, King Saud bin Abdulaziz University for Health Sciences, Riyadh, Saudi Arabia; Methodist University Cape Fear Valley Health School of Medicine, UNITED STATES OF AMERICA

## Abstract

**Background:**

Innovative modalities have been administered in educating Anatomy to medical undergraduates, specifically Neuroscience because of its clinical significance. For building strong foundation in Anatomy, focus is shifting towards adaptation of digital resources for delivery of content such as three-dimensional (3D) digital resources like 3 D Sectra Visualization Table (SVT). However, lack of dimensional details and relationship of displayed Neuroanatomy images and limited applicability of its digital dissection features necessitate an adjunct teaching strategy to complement the deficiencies in Sectra visualization table. Therefore, to bring about meaningful learning in Neuroanatomy, a model of flipped classroom was designed with existing three-dimensional digital Anatomy resource (3D SVT) and observed the comparison between two approaches on students’ academic performance.

**Objectives:**

The study was designed to accomplish the following two objectives: To compare the effect of flipped classroom teaching strategy combined with a 3 D digital Anatomy resource (SVT) and 3D digital Anatomical resource (SVT) alone on students academic performance in Neuroanatomy through pre and post-test scores To assess medical students’ perceptions of the flipped classroom model integrated with digital anatomy resources in the context of learning Neuroanatomy.

**Methods:**

A Quasi- experimental study was conducted on two thirty-three medical students. Second year MBBS students were randomly divided into two groups. Group I (Experimental group) was exposed to the flipped classroom with digital anatomical resource while Group II (Control group) was taught through the 3D Sectra session. A pre and the posttest was held before and after each session. A five‐point Likert scale questionnaire was administered to evaluate students’ perception of the flipped classroom integrated model with Sectra (3 D digital resource). In the present study pre and post test scores of students for two groups were reported, within group comparison was made using Wilcoxon sign rank test and between group comparison was done using Mann Whitney U test. Comparison of test scores was also done with respect to gender.

**Results:**

The results of the study revealed that after exposure to flipped classroom with 3D digital resource, a significant increase in the posttest score of interventional Group was observed in comparison to control Group post test score after receiving session with 3D resource alone (p < 0.001). However, with regards to gender, both male and female students give an average similar performance giving no significant difference in their performance. The reliability coefficient for perceptions of experimental group students after exposure to flipped model showed the overall Cronbach’s alpha of 0.94.

**Conclusion:**

The study showed the flipped classroom with 3D digital resource approach significantly improved students’ test scores compared to the sessions with 3D resource alone. Moreover, a general positive perception of the new teaching approach was reported by students of flipped classroom.

## Introduction

### Background

In basic medical sciences, dissection has been acknowledged as an ideal methodology for learning Anatomy. However, with commencement of integrated curriculum in Medicine it is now obsolete [1,2]. To accomplish academic aims and to address the changes in curriculum, adaptation of range of digital technologies has been embraced in Modern Anatomy teaching. New modalities have been applied in teaching Anatomy like Digital Anatomical resources, such as virtual reality, augmented reality and Sectra visualization table (SVT) through which three-dimensional (3D) viewing of human body is possible [1,3]. Among them SVT is a 3D digital tool used for teaching Anatomy through a touch screen with features of digital dissection [4]. Though having dissection features, restricted feasibility in terms of regional selection and its smaller scale in comparison to other products which have availability of virtual dissection of full human body, has limited its applicability [5]. Beside Sectra, even 3D virtual models currently available inaccurately represent relationships between organs [6]. Therefore the desired effects of these digital Anatomy teaching modalities are still in controversy [7,8].

In addition, there is a risk of engagement of students in fruitless learning strategies and loss of new technology’s potential in case of failure of the faculty to plan suitable learning activities using three-dimensional technology [9]. A Randomized control study conducted revealed no difference on learning ease through pre and posttest on comparing learning through 3D digital model (virtual reality) technology to online textbooks in the Neuroanatomy course. In the above-mentioned study despite evidence of perceptions, no significant difference was observed in summative examination performance between the groups studied; suggesting learning perceived by students might not be translated into retention of knowledge in the long term [10]. Moreover not more than 4–5 students can stand around the visualization table at one time which necessitates increasing the number of groups: presenting a logistical challenge in the curriculums of medical school [11].

Though improved understanding of clinically applied anatomy was reported by the students [12], a meta-analysis revealed a divergency about effectiveness of learning by 3D visualization in the subject of Anatomy [13]. Similarly another meta-analysis reported no statistically significant difference in undergraduates’ test performance who were taught Anatomy though three-dimensional virtual/augmented tools with control [8].

Hence To build a strong foundation of Anatomy, specifically higher degree of spatial complexity in Neuroanatomy which is considered difficult to teach by Anatomist and to learn by medical students, because of its various discrete structures [14, 15]. It is of utmost importance to explore creative, appealing and engaging multimodal means that promote proactive learning and long-term, effective retention of knowledge. While digital approaches are being adopted in Anatomical education, it is important to promote a more active learning strategy, not only calibrating content delivery versus the digital resources but also providing students and instructors with clear course alignment and learning objectives [16].

Usage of interactive teaching tools in combination would be considered as a solution to this problem. In the evolution of digital learning, success will be achieved by embracing resources with a smart learning environment and effective learning experience [5]. Integrated multimodal approaches might be a flexible mode for teaching Anatomy through combination of multiple instructional resources so as they complement one another [17].

In this regard a blended learning strategy can be considered that promotes active learning, the flipped classroom model, which is broadly defined as a teaching approach that reverses the traditional didactic teaching mode focusing on lecture style presentation to interactive activities in the classroom [16,18,19].

The flipped classroom is an educational paradigm that inverts traditional elements of lecture and homework in course design. It allows students to initially engage with the learning material before class, thus repurposing class time for active learning exercises in small groups, which facilitates a deeper understanding of the subject matter. This emphasis on student centered active learning fosters students’ motivation to acquire knowledge and enhance skills [20].

When teaching clinical Anatomy to medical students, the flipped classroom model was found to advance students to acquire basic knowledge in an active learning environment [21] and improve their performance by activating learning interest and cultivating their thinking ability [17,19].

To maximize meaningful learning of Neuroanatomy in undergraduate medical students because of this region’s clinical significance, faculty should modify their teaching strategies to fit the Undergraduate’s needs [22]. Though Sectra Table has a large library of 3D images covering all systems of the human body, its application is limited to selected regions. Especially in Neuroanatomy, there is a lack of three-dimensional details and relationship of displayed images and their cross sections at various levels, which are of real importance for meaningful learning of this region [5]. Moreover, there is limited feasibility of its digital dissection feature at a scale which is smaller as compared to other digital products like Anatomage Table, with facility of virtual dissection of full body [5]. This necessitates the use of teaching strategy which would complement 3D Sectra Digital resource to bring meaningful learning for medical undergraduates. There is a dearth of studies conducted directly comparing the scope of flipped classroom integrated with digital resource experience with students’ learning outcomes. Moreover, there is no comparison done with control group that was taught using the non-flipped “digital resource only teaching method” in parallel. Addressing this gap, the aim was to gather data (academic score) with digital resources alone and with complimentary teaching strategy, i.e., flipped classroom. The scarcity of robust quantitative studies was the rationale for this research with the opportunity to fill the gap in literature by evaluating learning outcomes with intention to contribute evidence-based practices.

### Significance of the problem

As digital resources are being implemented in the discipline of Anatomy, it is utmost significant to foster integrated teaching approaches like flipped class that will complement the digital tool, with intention of providing a meaningful learning outcome. Giving students with remote access to learning content in flipped class offers them the flexibility to learn at their own time and assists in managing cognitive load. Despite the popularity of the flipped classroom mode in higher education, there is limited evidence of these two approaches being combined and the utility of integrating flipped model of instruction with digital resource for imparting knowledge of Neuroanatomy. The results of this study would help in the execution of future Neuroanatomy courses in programs by identifying issues regarding the implementation of the blended integrating tools whereby Sectra would be used as adjunct tools to foster meaningful learning.

### Objectives of the study

The study was designed to accomplish the following two objectives:

To compare the effect of flipped classroom teaching strategy combined with a 3 D digital Anatomical resource (SVT) and 3D digital Anatomical resource (SVT) alone on students’ academic performance in Neuroanatomy through pre and post-test scores

To determine medical students’ perceptions of the flipped classroom model integrated with digital Anatomical resource in the context of learning Neuroanatomy

## Materials and methods

### Study design

Quasi- experimental study was conducted at the Department of Anatomy at Dow Medical College DMC, Dow University of Health Sciences DUHS. Two thirty-three Undergraduate second year medical students of Bachelor of Medicine Bachelor of Surgery program at DMC, DUHS in Neurosciences Module of Eight weeks starting from 24^th^ February to 22^nd^ APRIL, in the academic year of 2025 participated for the study after an approval from Institution Review Board IRB of DUHS.

A simple random sampling method was used to group the students into Group I and Group II. The purpose of the study was explained to the students, and an informed written consent duly signed by students was taken. Anonymity and confidentiality were maintained through collection, analysis and reporting of the data.

### Data collection procedure

A study population of second year medical students of MBBS program was included in this study. The effectiveness of flipped classroom was investigated in the module of Neurosciences in 2^nd^ year MBBS. At Dow Medical College, generally Anatomy is taught through lectures, practical, CBL and Sectra sessions. No flipped classroom sessions were ever taken in the MBBS curriculum, more specifically in the first two years of the program. After informed consent, Group I (Interventional group) was exposed to the flipped classroom with digital Anatomical resource (SVT) while Group II (Control group) was taught through the 3D Sectra Visualization table session only. Both groups were taught by the same facilitator. The learning objective of both strategies was also the same. A pre and post-test, each containing ten multiple-choice questions, was administered for assessing improvement and comparison of both teaching strategies.

#### Flipped sessions with digital anatomical resource (SVT).

Interventional group I (137 students) was exposed to flipped classroom lessons. In each session three stages were followed: Stage-1: pre-class; Stage-2: in-class; Stage-3: after-class.

For the pre‐class activities contents of the core topics was created as recordings of video lecture beforehand which were devised using images from Atlas of Netter, and online resources. Lectures were recorded using Screencast-O Matic web launcher v2.21.1 (JRE14) and PowerPoint 365. The lecture recording was 20–30 min in length. One week before the in-class session, the lecture recordings were uploaded on the online learning management system Moodle, version 4.0 and were made available to students for watching. Students were able to download video lectures from LMS onto their computers or see the recordings on LMS directly at their feasible time and venue. To ensure that students come prepared for class, a pretest was conducted to find out whether students had gone through the pre-class learning material.

For the in-class stage, interventional group was divided into three subgroups. Each subgroup received a session of one and half hour duration in neurosciences module. For interventional groups there were 3 sessions in total. For the in-class session, a touch screen visualization table, Sectra table (Linköping; Sweden) was used. It’s the F18 model of Sectra, with a sixty-five-inch monitor and has 4K resolution for viewing three-dimensional images of the Anatomical structures of the human body.

The students in the subgroup worked in 4 pairs at each Sectra visualization table at Dissection Hall of Dow Medical College. (There are 4 Sectra tables available at dissection Hall of Dow Medical College) For the in-class activities, with the cadaveric dissector tool on Sectra 3 D visualization table and 3D images of Neuro Anatomy, discussion was generated. Student-centered activities were designed including demonstrations and presentations on 3D models on screen by the students. The facilitator was on hand to help students in case of problems navigating the table and resolving any queries. Students were asked to explore the images using the visualization table.

For the After-class stage, a posttest consisting of ten Multiple-Choice Questions was provided for assessment in the after-class stage in each session. The perception of students regarding flipped classrooms incorporated with 3 D digital resources was assessed quantitatively through a pre- validated 30 items questionnaire on a Likert scale. A 5-point Likert scale with scores of 1–5 like 1: Strongly disagree (SDA), 2: Disagree (D), 3: Neither agree nor disagree (N), 4: Agree (A) and 5: Strongly agree (SA) was applied to observe the rating from the students. The survey consisted of two parts: the 1st part included demographic information, such as gender, while the 2nd part included the students’ perception of flipped classroom. Reliability of survey questionnaire through Pearson correlation coefficient was 0.87, indicating a high degree of reliability. It was given to the students at the end of each session [23].

#### 3D Sectra visualization table sessions.

Control group II (96 students) was exposed to 3D Sectra Visualization table session only. Like Experimental group, they were also divided into 3 subgroups each subgroup has session on Sectra for one and half hour duration on 3 separate days. In total there were 3 sessions of Sectra for control group. A pretest was conducted to find out the prior knowledge students had. For the Sectra session, a touch screen visualization table, Sectra table (Linköping; Sweden) was used to explore displayed 3D images of the human body. In each subgroup students were asked to work in 4 pairs at each Sectra visualization table at Dissection Hall of Department of Anatomy DMC.

Using cadaveric dissector tool on Sectra 3 D visualization table and 3D images of Neuro Anatomy, Student-centered activities were designed which included demonstrations and presentations on these digital tables by the students. The same facilitator who conducted flipped session was on hand to help students with navigation of the table and to resolve queries. Students then explored and discussed the images using the Sectra.

After each Sectra session, a post test consisting of ten Multiple-Choice Questions was provided for assessment to each control subgroups at the end of each session.

### Data analysis procedure

Data was stored and analyzed using IBM-SPSS version 23.0. Counts with percentages were reported on gender distribution with groups and the association was tested using Pearson Chi Square test. Median with Interquartile ranges were given on pre and post test scores. Normality of test scores was assessed using Kolmogorov Smirnov and Shapiro Wilk test of normality Comparison of test scores between groups was made using nonparametric Mann Whitney U test and Wilcoxon Sign Rank test was used to compare the pre and post test scores within groups. Stratification was also done with gender. Descriptive correct and incorrect responses on each item of test were also reported and comparison was made. For perception on flipped classroom, counts with percentages were given from experimental group after intervention. Means perceptions were also compared among gender using independent sample t-test. P-values less than 0.05 were considered statistically significant.

## Results

Out of 233 students, Group I, the interventional group, had 137 students (58.7%) while group II had 96 students (41.2%).

### Gender distribution in Group I and Group I

Table 1 reports the distribution of gender between two studied groups. In interventional Group, male students were 33 (24.1%) and females were 104 (75.9%), whereas in group –II male students were 33 (34.4%) and female were 63 (65.5%). The Pearson Chi Square test did not give any significant association of gender distribution with groups (p = 0.086).

**Table 1:** Distribution of gender between study groups.

*Gender*	*Interventional Group I* *(n = 137)*		*Control Group II* *(n = 96)*		*p- value*
	*n*	*%*	*n*	*%*	
***Male***	33	24.1	33	34.4	0.086
***Female***	104	75.9	63	65.6	

*p-value was obtained using Pearson Chi Square test.

### Comparison of test scores between groups

Results showed that before intervention in Group-I, median for test scores was 4 with Interquartile ranges (IQR = 2), and in Group-II, median for Pretest scores was 4.5 with (IQR = 01), whereas after intervention in Group –I, median for Test scores was 7 with (IQR = 2), and in Group-II median for Posttest scores was 4(IQR = 1). Mann Whitney U test did not give any significant median difference for pretest scores between two groups (p = 0.85), however, after intervention students in Group-I had higher median test scores which is significant in comparison to Group-II post test score (p < 0.001).Table 2 shows a comparison of differences in pre and posttest scores between two studied groups

**Table 2:** Comparison of test scores among groups.

*Test Scores*	*Group-I* *(n = 137)*			*Group-II* *(n = 96)*			*p- value*
	*Q1(25*^*th*^ *percentile)*	*median*	*Q3(75*^*th*^ *percentile)*	*Q1(25*^*th*^ *percentile)*	*median*	*Q3(75*^*th*^ *percentile)*	
***Pre***	4.00	4.00	6.00	4.00	4.50	5.00	0.85
***Post***	6.00	7.00	8.00	4.00	4.00	5.00	<0.001*

*p < 0.05 was taken Statistically Significant using Mann Whitney U test

### Comparison of Group-I test scores with gender before and after intervention

Table 3 reports the comparison of test scores between male and female students from Group-I at pre and post intervention. Results showed before intervention among male students, median for test scores was 4 with (IQR = 2), and median for female students was 4 with (IQR = 2), whereas after intervention among male students, median for Test scores was 7 with (IQR = 1), and among female median for test scores was 7(IQR = 2). Mann Whitney U test did not give any significant median difference between pre and post test score among male and female students of Group –I (p > 0.05).

**Table 3:** Comparison of Group-I test scores with gender before and after intervention.

*Test Scores*	*Group-I*						*p- value*
	*Male* *(n = 33)*			*Female* *(n = 104)*			
	*Q1(25*^*th*^ *percentile)*	*median*	*Q3(75th percentile)*	*Q1(25*^*th*^ *percentile)*	*median*	*Q3(75th percentile)*	
***Pre***	3.00	4.00	5.00	4.00	4.00	6.00	0.11
***Post***	6.00	7.00	7.00	6.00	7.00	8.00	0.056

* p < 0.05 was taken Statistically Significant using Mann Whitney U test.

### Comparison of Group-II test scores with gender

Table 4 reports the comparison of pre and posttest scores between male and female students from group-II, results showed among male students, median for pretest scores was 5 with (IQR = 2), and median for female students was 4 with (IQR = 1), whereas after Sectra session, among male students, median for posttest scores was 4 with (IQR = 1), and among females, median for test scores was 4(IQR = 1). Mann Whitney U test did give significant median difference for pretest scores of group-II male and female students (p = 0.01), but no significant median difference observed between male and female students of group-II in posttest score (p = 0.36).

**Table 4:** Comparison of Group-II test scores with gender.

*Test Scores*	*Group-II*						*p- value*
	*Male* *(n = 33)*			*Female* *(n = 63)*			
	*Q1(25*^*th*^ *percentile)*	*median*	*Q3(75th percentile)*	*Q1(25th percentile)*	*median*	*Q3(75th percentile)*	
***Pre***	4.00	5.00	6.00	4.00	4.00	5.00	0.01*
***Post***	4.00	4.00	5.00	4.00	4.00	5.00	0.36

* p < 0.05 was taken Statistically Significant using Mann Whitney U test.

### Comparison of test scores within groups

Table 5 reports the comparison of pre and posttest scores within study groups. Results showed that in group-I before intervention, median for pretest scores was 4 with (IQR = 2), and after intervention median for Posttest scores was 7 with (IQR = 2). However, in group –II students median for pretest scores was 4.5 with (IQR = 1) whereas median for posttest scores was 4(IQR = 1). The Wilcoxon Sign rank test showed that in Group-I median scores were significantly increased after intervention (p < 0.001), however no significant difference in median test scores of group-II students at pre and post stage (p = 0.74).

**Table 5:** Comparison of pre and post test scores within groups.

*Test Scores*	*Pre*			*Post*			*p- value*
	*Q1(25th percentile)*	*median*	*Q3(75th percentile)*	*Q1(25th percentile)*	*median*	*Q3(75th percentile)*	
***Group-I*** ***(n = 137)***	4.00	4.00	6.00	6.00	7.00	8.00	<0.001*
***Group-II*** ***(n = 96)***	4.00	4.50	5.00	4.00	4.00	5.00	0.74

* p < 0.05 was taken statistically significant using Wilcoxon Sign Rank test.

### Comparison of test scores within groups with respect to gender

Table 6 reports the comparison of test scores within groups with respect to gender. As shown in table, in Group-I for male students before intervention, the median for test scores was 4 (IQR = 2), and after intervention it was 7 (IQR = 1), Wilcoxon sign rank test showed a significant increase in median test scores of male samples in group –I (p < 0.01). Similarly in Group-I for female students before intervention, the median for test scores was 4 (IQR = 2), and after intervention it was 7 (IQR = 1), Wilcoxon sign rank test showed a significant increase in median test scores of female samples in Group –I (p < 0.01). Whereas in group-II for males students, the median for pre test scores was 5 (IQR = 2), and for posttest it was 4 (IQR = 1), Wilcoxon sign rank test showed no significant difference in median test scores of male students in Group –II (p = 0.45), and for females students in Group II the median for pre test scores was 4 (IQR = 1), and for posttest it was 4 (IQR = 1). Wilcoxon sign rank test showed no significant difference in median test scores of female students in Group –II (p = 0.29).

**Table 6:** Comparison of test scores within groups with respect to gender.

*Test Scores*	*Gender*	*Pre*			*Post*			*p- value*
		*Q1(25th percentile)*	*median*	*Q3(75th percentile)*	*Q1(25th percentile)*	*median*	*Q3(75th percentile)*	
***Group-I*** ***(n = 137)***	***Male*** ***(n = 33)***	3.00	4.00	5.00	6.00	7.00	7.00	<0.001*
	***Female*** ***(n = 104)***	4.00	4.00	6.00	6.00	7.00	8.00	<0.001*
***Group-II*** ***(n = 96)***	***Male*** ***(n = 33)***	4.00	5.00	6.00	4.00	4.00	5.00	0.45
	***Female*** ***(n = 63)***	4.00	4.00	5.00	4.00	4.00	5.00	0.29

* p < 0.05 was taken Statistically Significant using Wilcoxon Sign Rank test

### Item analysis of Group – I and Group – II students’ pre and posttest

**Fig 1:**
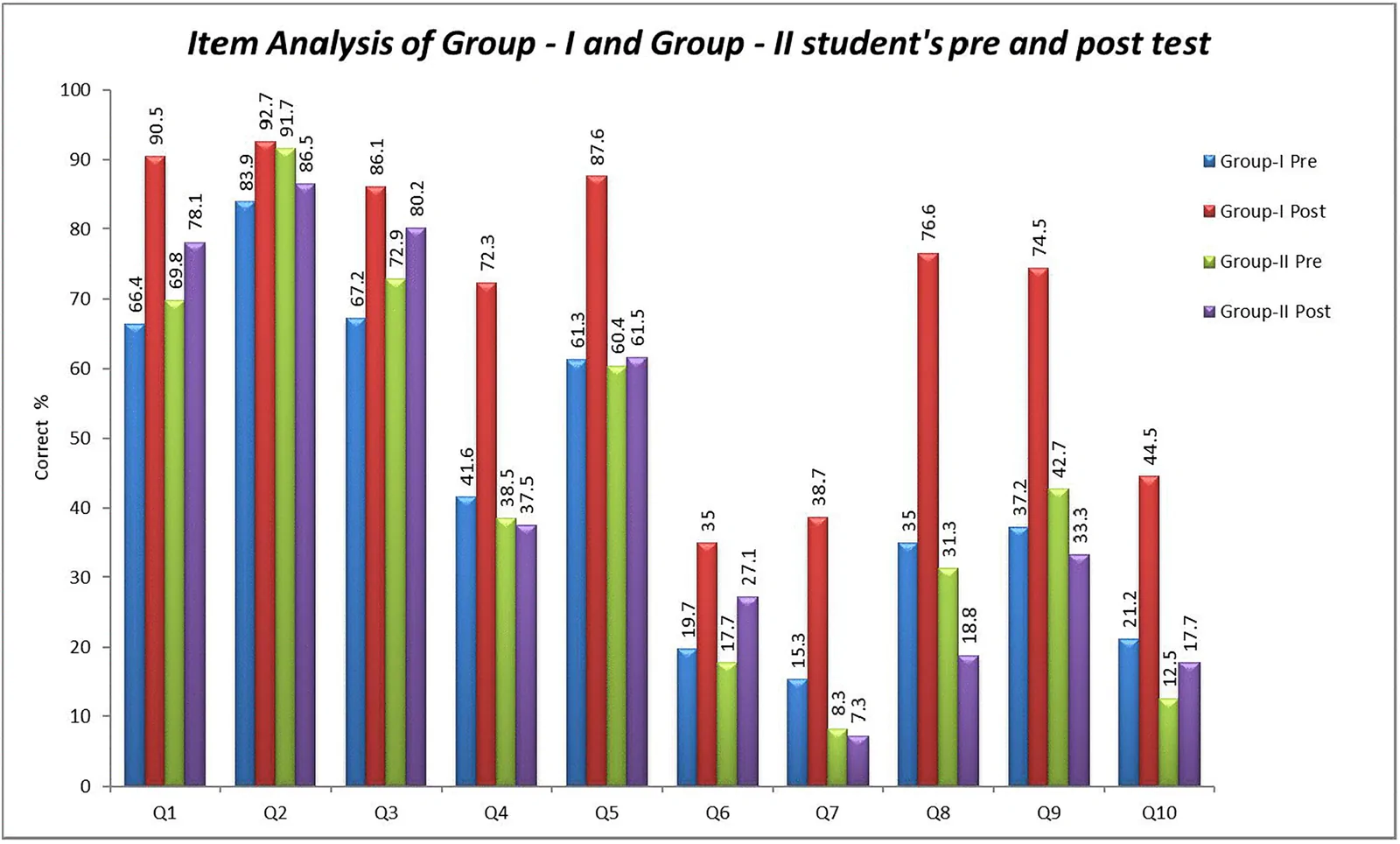
Item analysis of Group-I and Group-II students pretest and posttest.

Table 7 and Fig 1 reports the descriptive on each question for correct and incorrect responses from Group-I and Group-II students at pre and posttests.

**Table 7:** Item analysis of Group-I and Group-II student’s pre and posttest.

*Questions*		*Group – I* *(n = 137)*		*Group – II* *(n = 96)*	
		*Pretest* *n (%)*	*Post test* *n (%)*	*Pretest* *n (%)*	*Posttest* *n (%)*
***Q1***	***Incorrect***	46 (33.6)	13 (9.5)	29 (30.2)	21 (21.9)
	***Correct***	91 (66.4)	124 (90.5)	67 (69.8)	75 (78.1)
***Q2***	***Incorrect***	22 (16.1)	10 (7.3)	8 (8.3)	13 (13.5)
	***Correct***	115 (83.9)	127 (92.7)	88 (91.7)	83 (86.5)
***Q3***	***Incorrect***	45 (32.8)	19 (13.9)	26 (27.1)	19 (19.8)
	***Correct***	92 (67.2)	118 (86.1)	70 (72.9)	77 (80.2)
***Q4***	***Incorrect***	80 (58.4)	38 (27.7)	59 (61.5)	60 (62.5)
	***Correct***	57 (41.6)	99 (72.3)	37 (38.5)	36 (37.5)
***Q5***	***Incorrect***	53 (38.7)	17 (12.4)	38 (39.6)	37 (38.5)
	***Correct***	84 (61.3)	120 (87.6)	58 (60.4)	59 (61.5)
***Q6***	***Incorrect***	110 (80.3)	89 (65)	79 (82.3)	70 (72.9)
	***Correct***	27 (19.7)	48 (35)	17 (17.7)	26 (27.1)
***Q7***	***Incorrect***	116 (84.7)	84 (61.3)	88 (91.7)	89 (92.7)
	***Correct***	21 (15.3)	53 (38.7)	8 (8.3)	7 (7.3)
***Q8***	***Incorrect***	89 (65)	32 (23.4)	66 (68.8)	78 (81.3)
	***Correct***	48 (35)	105 (76.6)	30 (31.3)	18 (18.8)
***Q9***	***Incorrect***	86 (62.8)	35 (25.5)	55 (57.3)	64 (66.7)
	***Correct***	51 (37.2)	102 (74.5)	41 (42.7)	32 (33.3)
***Q10***	***Incorrect***	108 (78.8)	76 (55.5)	84 (87.5)	79 (82.3)
	***Correct***	29 (21.2)	61 (44.5)	12 (12.5)	17 (17.7)

### Comparison of the correct responses in pre and posttest within group

Table 8 reports the comparison of each question for correct responses in Group – I and Group -II students’ pre and posttest. As shown in table, among Group-I students, there was significant increase in proportion of the correct responses for all ten questions after intervention (p < 0.05), whereas in Group-II there was only significant difference observed for question number 6 and question number 8 responses in posttest. (p < 0.05).

**Table 8:** Comparison of correct responses in pre and posttest within group.

*Questions*	*Group-I* *Pretest vs. Posttest*		*Group-II* *Pretest vs. Posttest*	
	*Z – value*	*p -value*	*Z -value*	*p -value*
***post Q1 - pre Q1***	−5.284	<0.01*	−1.789	0.074
***post Q2 - pre Q2***	−3.000	0.003*	−1.508	0.132
***post Q3 - pre Q3***	−4.459	<0.01*	−1.698	0.090
***post Q4 - pre Q4***	−5.824	<0.01*	−.180	0.857
***post Q5 - pre Q5***	−5.091	<0.01*	−.174	0.862
***post Q6 - pre Q6***	−3.772	<0.01*	−2.183	0.029*
***post Q7 - pre Q7***	−5.060	<0.01*	−.378	0.705
***post Q8 - pre Q8***	−7.181	<0.01*	−2.449	0.014*
***post Q9 - pre Q9***	−6.231	<0.01*	−1.406	0.160
***post Q10 - pre Q10***	−4.824	<0.01*	−1.213	0.225

*p < 0.05 was taken statistically significant using Wilcoxon Sign Rank test.

### Comparison of the correct responses in pre and posttest between groups

Table 9 reports the comparison of each question for correct responses between Group-I and Group-II students, for both pre and posttest. Results showed, for pretest there was no significant difference in the proportion of the correctly given responses for all ten questions between Group-I and Group-II students, however in posttest significant differences were obtained in the proportion of correctly given responses from Group-I students as compared to responses from Group-II student for Question number 1, 4, 5, 7, 8, 9 and 10 with p < 0.05 using Mann Whitney U test.

**Table 9:** Comparison of correct responses in pre and posttest between groups.

*Questions*	*Pre* *Group – I vs. Group – II*		*Post* *Group – I vs. Group – II*	
	*Z -value*	*p -value*	*Z -value*	*p -value*
Q1	−0.540	0.589	−2.630	0.009*
Q2	−1.729	0.084	−1.569	0.117
Q3	−0.939	0.348	−1.202	0.229
Q4	−0.468	0.640	−5.279	<0.01*
Q5	−0.138	0.890	−4.643	<0.01*
Q6	−0.383	0.702	−1.281	0.200
Q7	−1.589	0.112	−5.383	<0.01*
Q8	−0.602	0.547	−8.694	<0.01*
Q9	−0.841	0.400	−6.236	<0.01*
Q10	−1.707	0.088	−4.260	<0.01*

* p < 0.05 was taken statistically significant using Mann Whitney U test.

### Perceptions of the flipped classroom model integrated with digital anatomical resource in Group I

Table 10 reports the reliability coefficient for perceptions of students from Group-I after intervention showing the overall Cronbach’s alpha for 30-items was 0.94. The overall and sub domain’s reliability coefficients were statistically adequate except for Perception of Independence and Responsibility was found little lower.

**Table 10:** Reliability analysis for perceptions of Group-I students after intervention.

*Perceptions on*	*Number of Items*	*Cronbach’s α*
Perception of Interest and Motivation	07	0.85
Perception of Engagement and Collaboration	06	0.82
Perception of Learning Efficiency and Preparedness	09	0.85
Perception of Teaching Strategy and Suitability	05	0.83
Perception of Independence and Responsibility	03	0.21
Overall	30	0.94

#### Perception of interest and motivation.

**Fig 2:**
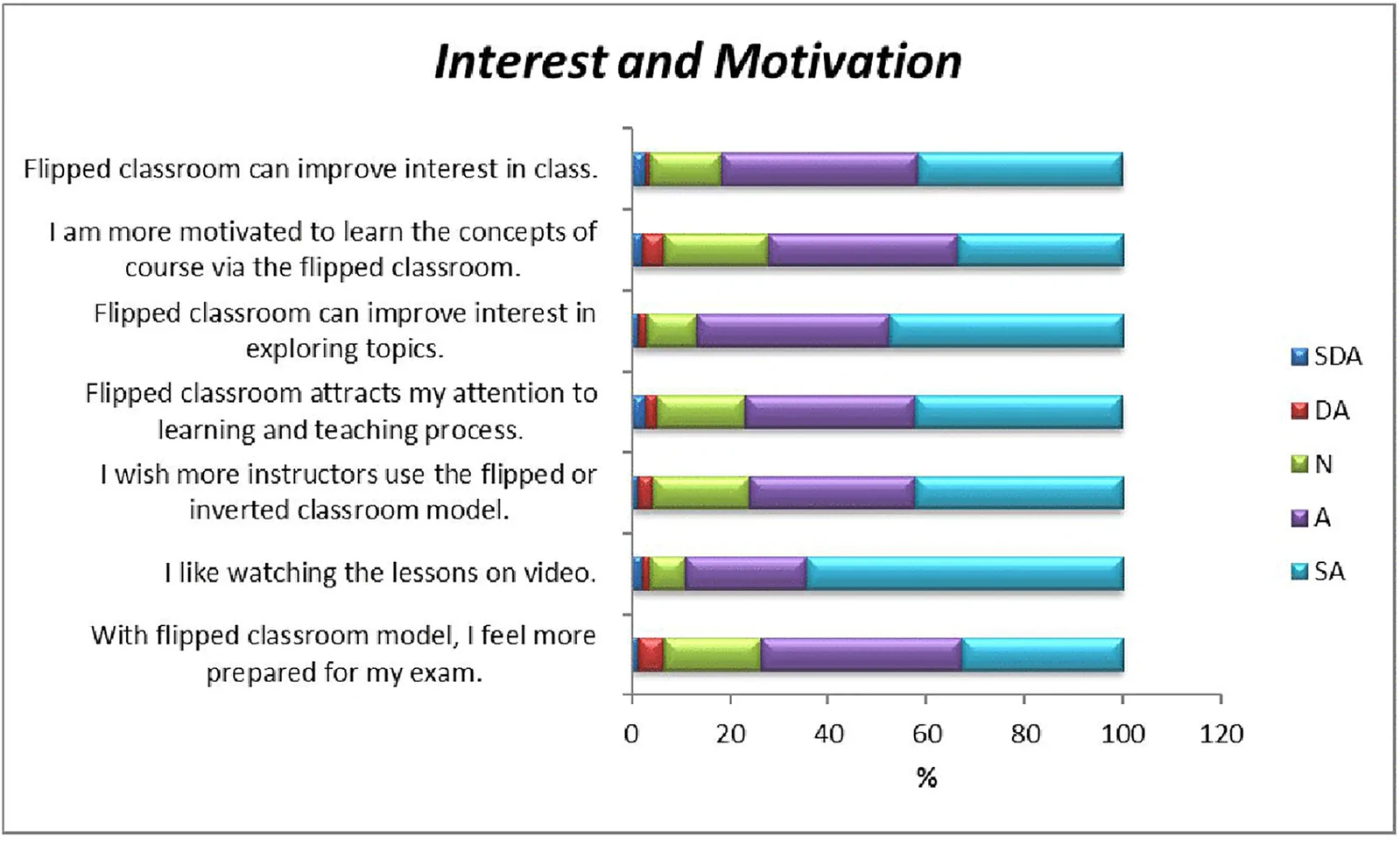
Perception of interest and motivation in flipped classroom.

Fig 2 reports the perception regarding interest and motivation of Group-I students after intervention. More than half of the students (73.7%) agreed or strongly agreeing on they felt more prepared. 89% liked watching the lessons on video. 75.9% wished more instructors would use that model, 76.6% think it attracts attention to learn and the teaching process. 86.8% believe this improves interest in exploring topics. 72.3% felt motivated to learn the concepts and 81.7% found it improving interest in class.

#### Perception on engagement and collaboration.

**Fig 3:**
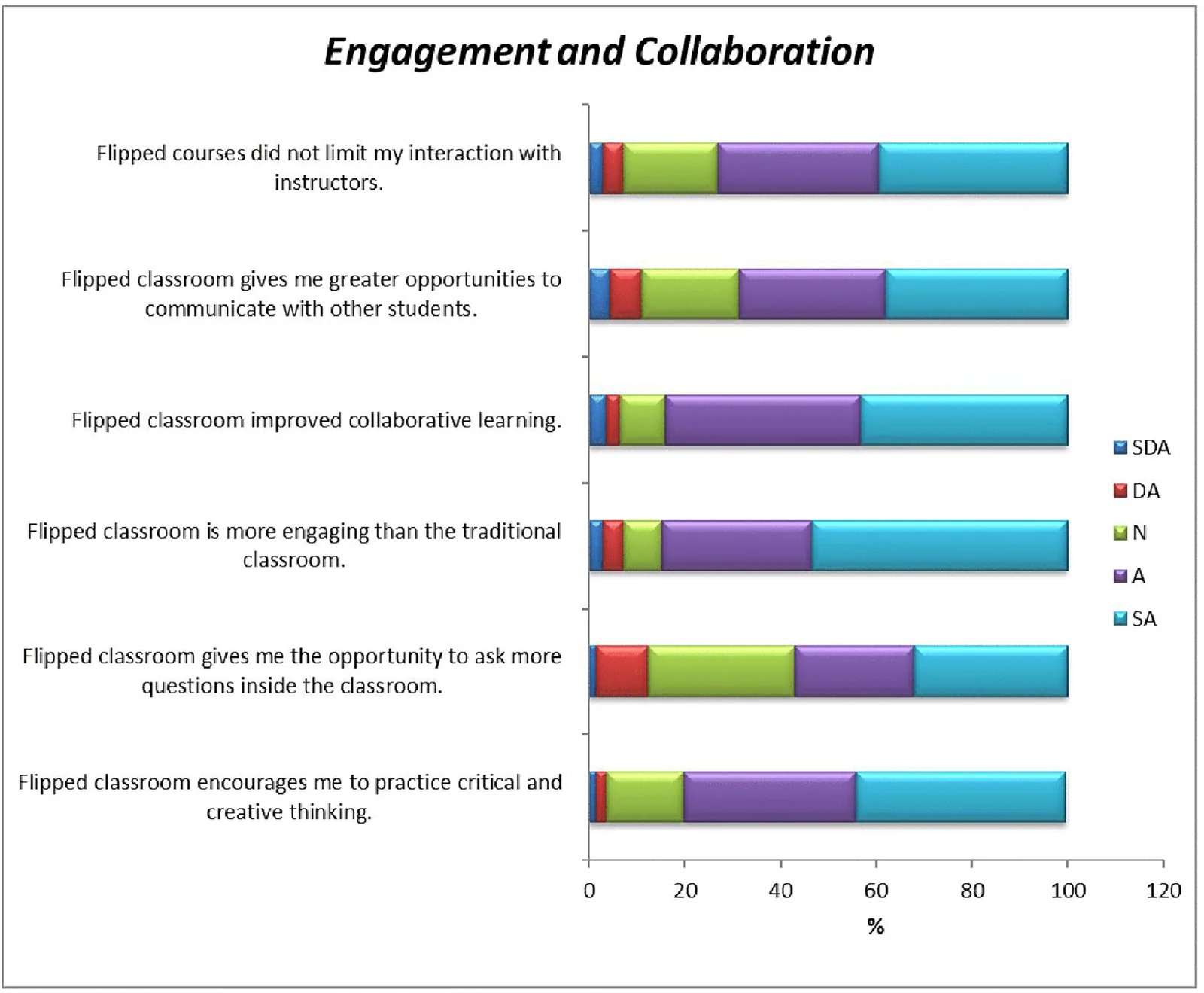
Perception of engagement and collaboration.

Fig 3 reports the perception regarding engagement and collaboration of Group-I students after intervention.80.3% were agreeing or strongly agreeing that the flipped class encourage them to practice critical and creating thinking, 56.9% thought that it gave opportunity for asking more questions in the class, 84.7% thought it to be engaging than traditional class, 84% believed that it improved collaborative learning,68.7% felt it gave them opportunities for communication with others greatly, and 73% felt their interaction with instructor was not limited.

#### Perception on learning efficiency and preparedness.

**Fig 4:**
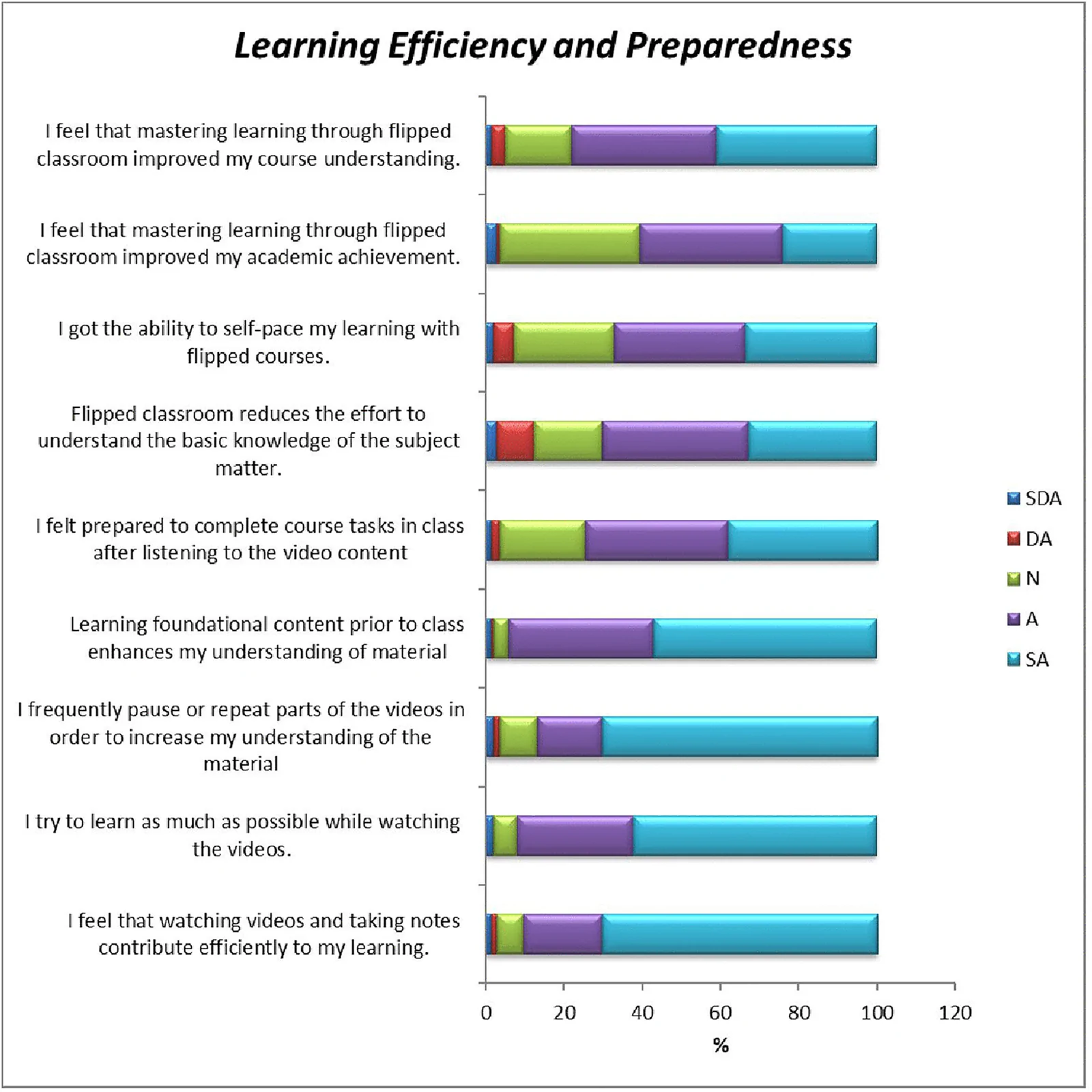
Perception on learning efficiency and preparedness.

Fig 4 reports the perceptions of Group-I students after intervention regarding learning efficiency and preparedness. 90.5% were agreeing or strongly agreeing on videos watching and note taking contributes them to learn efficiently, 91.9% tried as much as possible to learn while they were watching the videos, 86.9% frequently paused or repeated parts in videos to enhance understanding of the content, 94.1% thought learning foundation contents prior to class enhanced understanding of material, 74.5% felt that after going through video they were more prepared to finish the task given in class, 70% felt that the flipped class decreased their efforts to understand the basics of the subject content, 67.2% got the ability to self-pace their learning, 60.6% felt improved academic achievement was attained by mastering the learning through the flipped class, and 78.1% felt improved their course understanding by mastering the learning through flipped class.

#### Perception on teaching strategy and suitability.

**Fig 5:**
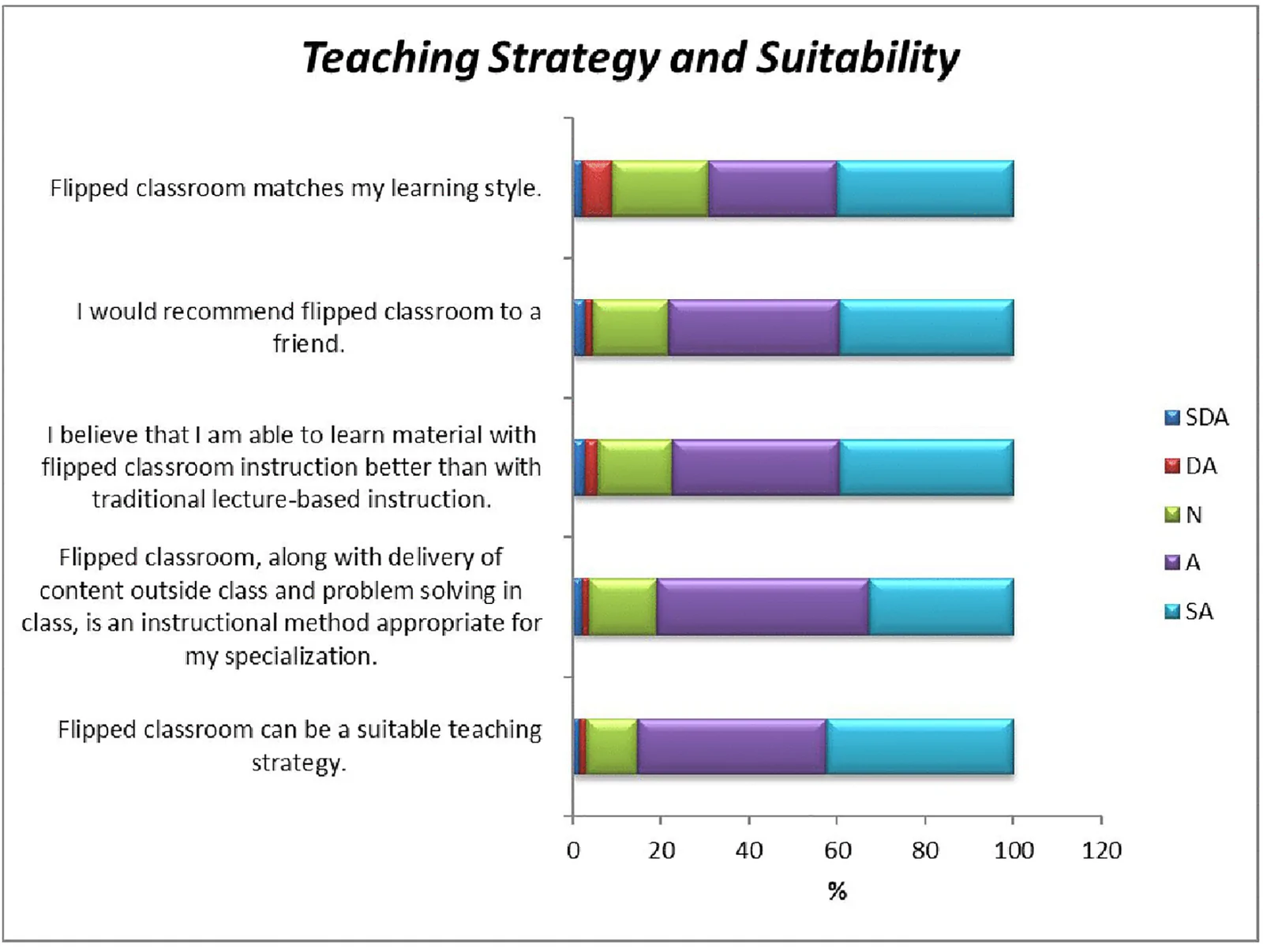
Perception on teaching strategy and suitability.

Fig 5 report the perception of students from Group-I after intervention on teaching and suitability, 85.4% were agreeing or strongly agree on flipped classroom suitability as a teaching strategy, 81% found it an appropriate instructional method for their specialization, 77.4% thought through the mode of flipped class, they learned well than traditional didactic teaching mode, 78.1% would recommend flipped mode to their friends, and for 69.3%, it matched their learning style.

#### Perception of independence and responsibility.

**Fig 6:**
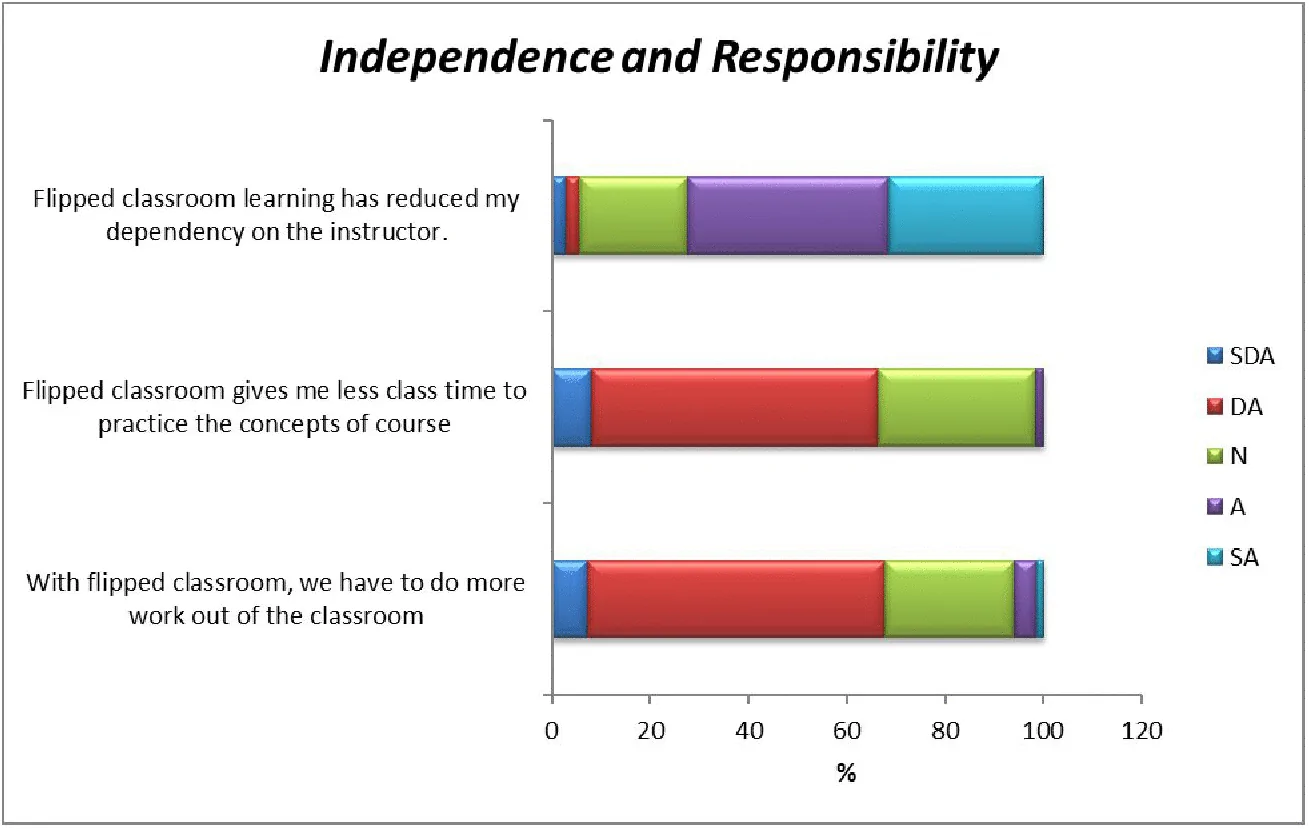
Perception of independence and responsibility.

Fig 6 reports the perception on independence and responsibility from Group-I students after intervention, 67.9% disagreed that they had to do work more out of the classroom in flipped instructional mode, 66.4% disagreed that it gave less in class duration to practice their concepts and 5.8% disagreed that it had reduced their dependency on the teacher.

### Mean comparison of perceptions with gender

Table 11 give the comparison of perceptions with respect to gender. Among male students, mean on perception regarding interest and motivation was 4.22 (SD=±0.56), regarding engagement and collaboration was 4.12 (SD=±0.70), regarding learning efficiency and preparedness was 4.20 (SD=±0.59), regarding teaching strategy and suitability was 4.21 (SD=±0.63) and regarding independence and responsibility was 2.86 (SD=±0.48), whereas among females, mean on perception regarding interest and motivation was 4.14 (SD=±0.70), regarding engagement and collaboration was 4.03 (SD=±0.75), regarding learning efficiency and preparedness was 4.20 (SD=±0.62), regarding teaching strategy and suitability was 4.06 (SD=±0.75) and regarding independence and responsibility was 2.84 (SD=±0.50). Independent sample t-test did not give any significant difference in the perceptions on flipped classroom between male and females (p > 0.05).

**Table 11:** Mean comparison of perceptions with gender.

*Domains*	*Gender*						*p-value*
	*Total*		*Male students*		*Female students*		
	*mean*	*S D*	*mean*	*S D*	*mean*	*S D*	
Perception of Interest and Motivation	4.16	0.67	4.22	0.56	4.14	0.70	0.56
Perception of Engagement and Collaboration	4.05	0.74	4.12	0.70	4.03	0.75	0.54
Perception of Learning Efficiency and Preparedness	4.20	0.61	4.20	0.59	4.20	0.62	0.95
Perception of Teaching Strategy and Suitability	4.09	0.73	4.21	0.63	4.06	0.75	0.28
Perception of Independence and Responsibility	2.85	0.49	2.86	0.48	2.84	0.50	0.87

* p < 0.05 was taken statistically significant using independent sample t-test.

## Discussion

The research study examined the effect of flipped instructional mode of teaching integrated with three-dimensional digital resources on improvement of academic scores and perception of undergraduate medical students in neurosciences module of anatomy. Using quantitative data, the effectiveness of flipped classroom, in contrast to digital resource session alone was evaluated. Along with scores, perceptions yielded another quantitative insight regarding the experience of students using flipped classrooms integrated with digital resources. There was dearth of evidence of such comparison in literature.

The findings of the study indicated that a flipped classroom model integrating three-dimensional digital resources has positively influenced students’ test scores suggesting it to be more effective than sessions using 3D digital resources alone.

Few of the studies which were conducted had several limitations. One of the relevant studies that could be found has a small sample size. However, due to absence of control group there was no direct comparison found about the extent of flipped model with 3D digital anatomical resources on students’ academic/ learning performances hence could not find the direct evaluation of such effectiveness of this paradigm integrated with digital resources [19].

The results of the study revealed that after exposure to flipped classroom with 3D digital resources, a significant increase in the posttest score of experimental Group was observed in comparison to control Group posttest score who received sessions with 3D resource alone. Likewise, a significant marked increase in marks of medical students in their end semester was seen in a study conducted in 2022 supporting the likeness that there would be improvement in understanding of anatomical content when digital resources are present to support the flipped paradigm. However, no control group was available in their study which was included in our study [19]. Matching their finding with ours could be due to the inclusion of senior students in their study who have higher level of self-efficacy in comparison to students at junior level [24]. Similarly, Thomas in 2024 observed significant improvement in students’ score in understanding of some complex concepts suggesting facilitation of deeper learning by using flipped approach with digital interactive resources [25].

Another study on comparing flipped group taught with a Human Anatomy web based learning system with a control group came to find a significant improved scores in anatomy implying positive outcome in learning among students of the former group [17] one more study reported the similar result which was conducted on nursing students with similar mode of instruction [26] In the anatomy course of dental students, higher grades were achieved in final exams after adopting flipped classroom model suggesting its role in learning progression [27].

However, there are some studies that revealed no significant difference in scores of students learning between flipped and traditional approach [28]. Along the line is a randomized control trial where no significant difference statistically was observed in grades of students who received flipped and those who don’t [29]. Reason could be their comparison with traditional lectures rather than digital resources or it could all depend on how the lesson was planned by the teacher or the lack of students’ self or independent learning abilities [30] which is the main supportive perception about flipped classroom [31].

Results of the flipped studies with in significant post score which are in contrast with our study could be due to the use of 3D digital tools in flipped models in our study as both virtual and augmented digital anatomical tools provide different experience to comprehend the complexities in learning anatomical structures [12], which allows learners to develop a deeper understanding of surface and internal anatomical structures in relation to their surroundings which leads to improved post scores [32].

No significant difference was observed in test scores of Group-II (control) students who were exposed to 3D digital resources alone. Two of the studies conducted by Moro in 2017 [33] and 2021 [8] similarly did not find improved scores in medical students’ test followed by digital anatomy teaching by augmented or virtual reality tools [8]. Likewise, another study could not find any association between the usage of digital application and test scores in Anatomy [34].

In a local study conducted in the same city also demonstrated no significant difference in scores of students who were exposed to Sectra with those taught through traditional method, which could be because of small sample size (no of students = 50) of their study [1].

In contrast, students’ performance was better in lab tests who make use of a virtual dissection table in their anatomy course in comparison to those using cadavers [35]. Hence establishing that for contextual understanding of structures in human body, it is necessary to be spatially aware of the human structures [19].

One of the positive outcomes of our study was students’ perception whereby the value of 3D digital resource integration in flipped model was appreciated in their learning experience. Positive influence on students’ perception was also exhibited in research regarding the value of digital tools in understanding Anatomy which could be due to sophisticated nature of these resources’ fidelities [19]. Generally positive perception was also reported in a study which found flipped classroom to be enhancing the learning experience by being more engaging and effective despite encountering difficulties [25].

However, in a study students preferred the traditional approach of instruction to flipped classroom which could be due to limited availability of time for them to go through pre class as they were not full-time students, eventually they were unable to manage added preparatory workload. Hence a contrast in perception was observed in it with other studies which have full-time students who have rather flexible schedules lead to effective engagement in flipped class [36].

Difference in perception could be attributed to multiple factors including academic status of students (junior vs senior students) or it might be digital tools’ technical fidelities and capacity on regional organs versus neuroanatomy or the duration the students spend in these digital sessions would influence their engagement and experience towards these digital instructional designs [19].

Gender might be another factor that affects perception about flipped mode of learning. Having significant perception overall in our study, no significant difference in the perceptions on flipped classroom between male and females were found in accordance there are many studies where both genders showed no difference in the conducted survey [37].

There are some contradictory findings observed in literature where perception on flipped was found to be significantly different between genders with male giving preference to flipped than females. This contradiction in that study could be associated with culture in which male students seem to be more active in their utilization of technology than female students [23].

## Conclusion

Study showed that flipped class integrated with three-dimensional anatomical digital resource has proved to be exhibiting positive influence on students’ test scores and perception.

## Supporting information

S1 File SPSS output of the statistical analyses. (SPV)

S2 File Excel file with the graphs and associated data. (XLSX)

S3 File SPSS dataset used for statistical analysis. (SAV)

S4 File Study questionnaire. (DOCX)

S5 File Inclusivity in Global Research questionnaire. (DOCX)

S6 File Minimum dataset in SPSS format. (SAV)
