# Supplementary material for: Integrating anatomy learning: A comparative study of anatomical digital resource and flipped model with sectra
Source: PLoS One. 2026 Aug 31;21(8):e0356693. doi: 10.1371/journal.pone.0356693 (PMC13529019; doi:10.1371/journal.pone.0356693)
Supplement: Supplementary file 1 [file pone.0356693.s004.docx]

**Appendix – I Questionnaire**

**Roll no: ________________**

**Gender: ______________**

These questions will have a five-point Likert scale

1: Strongly disagree, 2: Disagree, 3: Neither agree nor disagree, 4: Agree, 5: Strongly agree

| **No** | **Item** | **1** | **2** | **3** | **4** | **5** |
| --- | --- | --- | --- | --- | --- | --- |
| 1 | I feel that watching videos and taking notes contribute efficiently to my learning. |  |  |  |  |  |
| 2 | With flipped classroom model, I feel more prepared for my exam. |  |  |  |  |  |
| 3 | I like watching the lessons on video. |  |  |  |  |  |
| 4 | I try to learn as much as possible while watching the videos. |  |  |  |  |  |
| 5 | I wish more instructors use the flipped or inverted classroom model. |  |  |  |  |  |
| 6 | I frequently pause or repeat parts of the videos in order to increase my understanding of the material. |  |  |  |  |  |
| 7 | Flipped classroom encourages me to practice critical and creative thinking. |  |  |  |  |  |
| 8 | Learning foundational content prior to class enhances my understanding of material. |  |  |  |  |  |
| 9 | Flipped classroom gives me the opportunity to ask more questions inside the classroom. |  |  |  |  |  |
| 10 | Flipped classroom attracts my attention to learning and teaching process. |  |  |  |  |  |
| 11 | With flipped classroom, we have to do more work out of the classroom. |  |  |  |  |  |
| 12 | Flipped classroom can be a suitable teaching strategy. |  |  |  |  |  |
| 13 | Flipped classroom can improve interest in exploring topics. |  |  |  |  |  |
| 14 | I felt prepared to complete course tasks in class after listening to the video content. |  |  |  |  |  |
| 15 | Flipped classroom is more engaging than the traditional classroom. |  |  |  |  |  |
| 16 | Flipped classroom gives me less class time to practice the concepts of course. |  |  |  |  |  |
| 17 | Flipped classroom reduces the effort to understand the basic knowledge of the subject matter. |  |  |  |  |  |
| 18 | Flipped classroom, along with delivery of content outside class and problem solving in class, is an instructional method appropriate for my specialization. |  |  |  |  |  |
| 19 | I am more motivated to learn the concepts of course via the flipped classroom. |  |  |  |  |  |
| 20 | Flipped classroom improved collaborative learning. |  |  |  |  |  |
| 21 | Flipped classroom can improve interest in class. |  |  |  |  |  |
| 22 | I got the ability to self-pace my learning with flipped courses. |  |  |  |  |  |
| 23 | Flipped classroom gives me greater opportunities to communicate with other students. |  |  |  |  |  |
| 24 | I believe that I am able to learn material with flipped classroom instruction better than with traditional lecture-based instruction. |  |  |  |  |  |
| 25 | I would recommend flipped classroom to a friend. |  |  |  |  |  |
| 26 | Flipped classroom matches my learning style. |  |  |  |  |  |
| 27 | I feel that mastering learning through flipped classroom improved my academic achievement. |  |  |  |  |  |
| 28 | Flipped courses did not limit my interaction with instructors. |  |  |  |  |  |
| 29 | I feel that mastering learning through flipped classroom improved my course understanding. |  |  |  |  |  |
| 30 | Flipped classroom learning has reduced my dependency on the instructor. |  |  |  |  |  |
